# Plant-derived extracellular vesicles for intestinal mucosal repair: mechanisms, microbiota-host interactions, and bioengineered delivery

**DOI:** 10.3389/fbioe.2026.1997653

**Published:** 2026-10-23

**Authors:** Dianbo Dong, Xiaoyan Dong, Lina Zhang, Wei Liu

**Affiliations:** 1 Department of Anorectal Surgery, Liaocheng People’s Hospital, Liaocheng, Shandong, China; 2 Yanggu County Hospital of Traditional Chinese Medicine, Liaocheng, Shandong, China; 3 Department of Critical Care Medicine, Liaocheng People’s Hospital Affiliated to Shandong First Medical University, Liaocheng, Shandong, China; 4 Department of Neurosurgery, Liaocheng People’s Hospital Affiliated to Shandong First Medical University, Liaocheng, Shandong, China

**Keywords:** engineered delivery, exosome-like nanoparticles, gut microbiota, intestinal epithelial barrier, intestinal mucosal repair, plant-derived extracellular vesicles

## Abstract

Plant-derived extracellular vesicles (PDEVs) and related nanoparticle preparations are being investigated for intestinal mucosal repair. Much of the therapeutic evidence concerns heterogeneous plant-derived exosome-like nanoparticles (ELNs) isolated following plant tissue disruption; this review therefore distinguishes extracellularly released vesicles, tissue-derived nanoparticle preparations with unresolved origins, and reconstructed carriers. Building on foundational edible plant nanovesicle studies, we synthesize preclinical evidence across inflammatory control, structural repair, regenerative activity, and functional barrier recovery. Reported effects include modulation of macrophage activity, inflammasome signaling, and mucosal immune responses, reduced neutrophil-associated injury, epithelial protection, and improvements in junctional and mucus-associated barrier features. Intestinal stem cell responses and organoid formation support regenerative activity in selected studies, although durable regeneration remains insufficiently established. Uptake of plant-derived RNAs by gut microorganisms and metabolic relays involving tryptophan derivatives and short-chain fatty acids connect selected preparations to microbiota-host communication. Engineering strategies support drug and nucleic acid delivery, gastrointestinal protection, local colonic exposure, and cellular uptake. Evidence remains predominantly preclinical, with gaps in preparation identity, batch consistency, causal validation of active cargo, dose-exposure-response relationships, safety, and scalable manufacturing. Translation requires preparation-specific characterization and reproducible potency assays, together with integrated assessment of mucosal structure, regeneration, barrier function, and durability.

## Graphical Abstract

**Figure:**
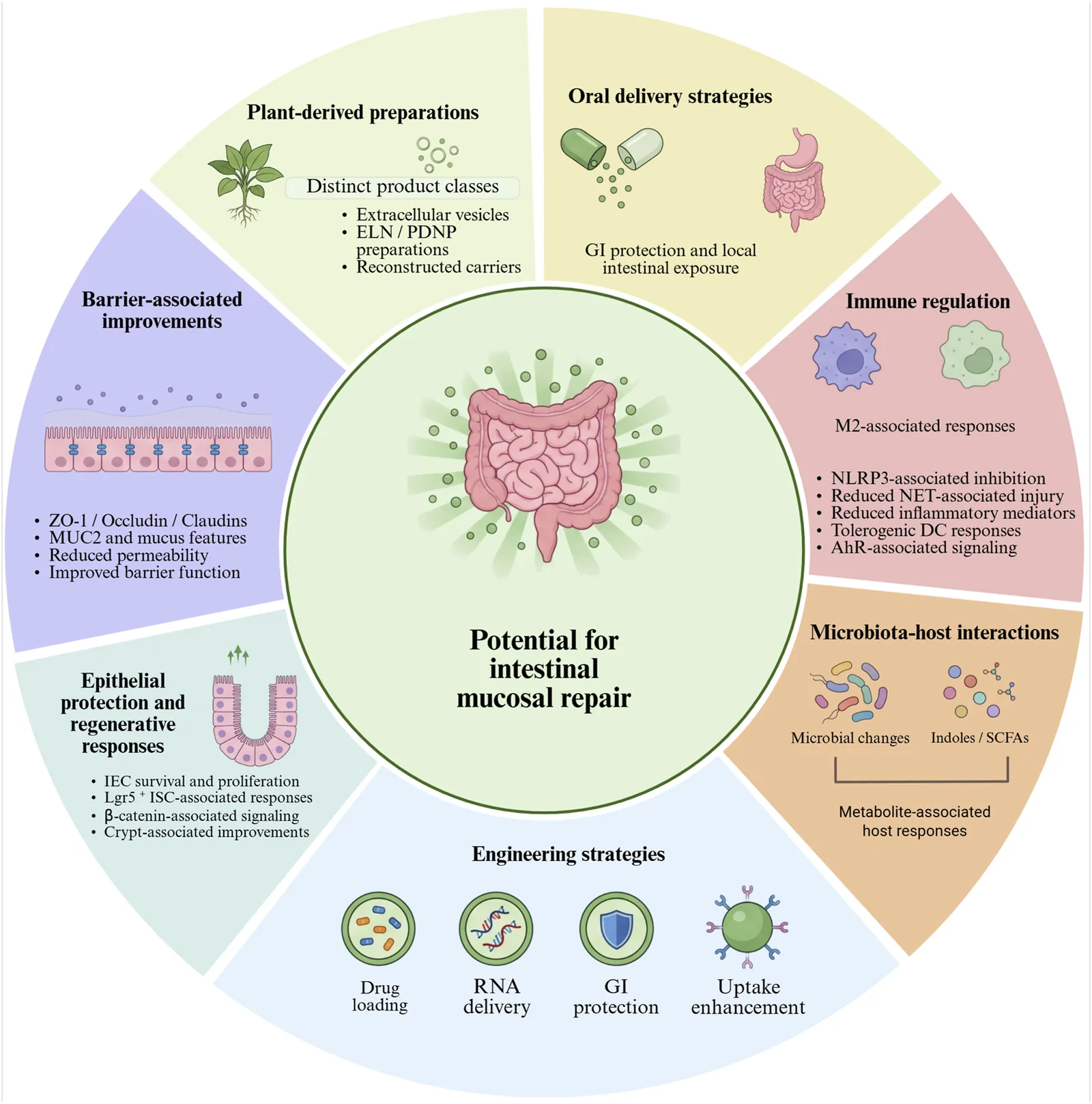
Conceptual overview of plant extracellular-vesicle research and related nanoparticle preparations in intestinal mucosal repair. The schematic integrates immune regulation, microbiota-host communication, epithelial protection and regeneration, barrier-associated effects, and engineered delivery. It summarizes representative preclinical findings across different preparations and does not imply that every material is a verified extracellular vesicle or that every study establishes complete functional mucosal recovery. Created in BioRender. liu, W. (2026) https://BioRender.com/48a7tt8.

## 1 Introduction

Intestinal mucosal homeostasis depends on coordinated interactions among intestinal epithelial cells (IECs), intercellular junctions, mucus, antimicrobial molecules, mucosal immunity, intestinal stem cells (ISCs), and the gut microbiota (Yao et al., 2024; Okumura and Takeda, 2018). Barrier disruption permits microbial access to mucosal immune compartments, triggering inflammation, oxidative stress, and neutrophil-associated injury that can further damage the epithelium and stem-cell niche (Barbara et al., 2021; Ortega-Zapero et al., 2025). These processes occur in inflammatory bowel disease (IBD) and other forms of intestinal injury (Selvakumar and Samsudin, 2025). Persistent defects in barrier integrity, epithelial regeneration, and microbiota–immune homeostasis can compromise disease control and long-term outcomes.

Therapeutic goals in intestinal disease extend beyond symptom control and inflammatory suppression toward mucosal healing. Emerging therapies should therefore be assessed separately for inflammatory control, structural repair, regenerative activity, and functional barrier recovery (Neurath and Vieth, 2023; Liu et al., 2021). Reduced inflammation may coexist with persistent permeability defects or inadequate epithelial renewal, supporting approaches that combine immune regulation with tissue repair (Liu et al., 2021; Ho et al., 2020).

Clinical assessment of mucosal healing combines disease-specific endoscopy, symptoms, and biomarkers, which provide complementary information and should not be treated as interchangeable endpoints. The Mayo endoscopic subscore (MES) and Ulcerative Colitis Endoscopic Index of Severity (UCEIS) are used in ulcerative colitis, while the Simple Endoscopic Score for Crohn’s Disease (SES-CD) assesses endoscopic disease activity in Crohn’s disease. Fecal calprotectin and C-reactive protein support monitoring, but biomarker normalization alone does not establish endoscopic healing. STRIDE-II identifies endoscopic healing as a long-term treatment target; histological healing in ulcerative colitis and transmural healing in Crohn’s disease provide additional measures of remission depth rather than universal formal treatment targets (Neurath and Vieth, 2023; Turner et al., 2021). Experimental repair assessment should likewise distinguish junctional proteins, mucus and crypt architecture, epithelial/ISC responses and organoid formation, and functional measures such as transepithelial electrical resistance (TEER), intestinal permeability, and microbial translocation (Liu et al., 2021; Vieujean et al., 2026; Rath et al., 2023). Integrating these complementary endpoints can help connect experimental mechanisms to clinically meaningful recovery and patient-centered treatment goals (Long, 2025).

Plant-derived vesicular preparations may combine local bioactivity with gastrointestinal delivery, although stability and targeting are preparation-dependent (Fang and Liu, 2022; Han et al., 2025; Li et al., 2025a; Alsaid et al., 2026). Pioneering grape, grapefruit, and broccoli studies by Huang-Ge Zhang and colleagues established interconnected directions in ISC responses, macrophage targeting, and dendritic-cell (DC) AMP-activated protein kinase (AMPK) signaling (Ju et al., 2013; Wang et al., 2014; Deng et al., 2017). Complementary ginger research by Mingzhen Zhang, Didier Merlin, and colleagues demonstrated anti-inflammatory and epithelial-protective activity (Zhang et al., 2016). These contributions underpin subsequent work on functional cargo and engineered delivery (Xu et al., 2026); their historical development is summarized in Section 2.1 and Table 1.

**TABLE 1:** Historical milestones in edible plant-derived nanovesicle research.

Year/journal	Study and plant platform	Key discovery	Scientific significance	Refs.
1967 Journal of Ultrastructure Research	Halperin and Jensen Carrot cell cultures	Ultrastructural observations of multivesicular bodies and vesicle release into the cell-wall space	Early structural context for plant vesicle release; distinct from later edible-plant therapeutic research	(Halperin and Jensen, 1967)
2013 Molecular Therapy	Ju et al. Grape GELNs	Lgr5^+^ ISC responses, organoid formation, and protection in DSS-colitis mice	Early proof linking edible plant nanoparticles to mammalian stem-cell biology and intestinal tissue regeneration	(Ju et al., 2013)
2013 Nature Communications	Wang et al. Reconstructed grapefruit-lipid nanovectors	Plant lipids reassembled into carriers for therapeutic cargo and targeted delivery	Established an early engineering approach; reconstructed carriers are distinct from native vesicles	(Wang et al., 2013)
2014 Molecular Therapy	Wang et al. Grapefruit GDNs	Oral macrophage targeting, HO-1 induction, colitis protection, and methotrexate delivery	Connected intrinsic intestinal immune targeting with *in vivo* therapeutic activity and oral drug delivery	(Wang et al., 2014)
2016 Biomaterials	Zhang et al. Ginger GDNPs	Gastrointestinal compatibility, anti-inflammatory activity, and epithelial survival/proliferation	Expanded orally administered plant nanomedicine toward epithelial protection and repair	(Zhang et al., 2016)
2017 Molecular Therapy	Deng et al. Broccoli BDNs	DC targeting and sulforaphane-associated, AMPK-dependent immune modulation	Extended immune targeting to antigen-presenting cells with DC-transfer and genetic mechanistic evidence	(Deng et al., 2017)
2018 Cell Host & Microbe	Teng et al. Ginger ELNs and other edible plant preparations	Microbial RNA transfer, altered bacterial gene expression, and a tryptophan-metabolite relay	Established a cross-kingdom microbiota-host mechanism that broadened the field beyond mammalian-cell targeting	(Teng et al., 2018)

The 1967 carrot-cell study provides early ultrastructural context; the subsequent entries are representative therapeutic milestones, not an exhaustive priority list. The grapefruit 2014 study combines therapeutic and immune-targeting contributions in one entry. Dates refer to journal publication year. Original particle designations are retained without assuming extracellular biogenesis. ISC, intestinal stem cell; DC, dendritic cell. The grape, grapefruit, broccoli, and microbial-RNA milestones include work by Huang-Ge Zhang and colleagues; the ginger milestone is the complementary work of Mingzhen Zhang, Didier Merlin, and colleagues

This review considers plant-derived extracellular vesicles (PDEVs), related exosome-like nanoparticles (ELNs), plant-derived nanoparticle (PDNP) preparations, and engineered carriers because these materials underpin much of the intestinal therapeutic literature. Original study designations are retained, but inclusion does not establish extracellular biogenesis or justify attributing tissue-derived particle or reconstructed-carrier effects to *bona fide* PDEVs (Welsh et al., 2024; Lian et al., 2022).

Existing reviews have addressed intestinal homeostasis, microbiota regulation, and plant-derived drug delivery (Li et al., 2025a; Yang J. et al., 2025; Tan et al., 2026). Here, we examine how immune regulation, epithelial protection, ISC-associated responses, microbiota–host interactions, and engineering contribute to mucosal repair. We distinguish disease amelioration, inflammatory control, structural repair, regenerative activity, and functional recovery according to the endpoints measured, and identify requirements for reproducible translation.

## 2 Biological features of plant extracellular vesicles and related nanoparticle preparations

### 2.1 Historical development, definition, nomenclature, and sources

#### 2.1.1 Historical background and milestones in edible plant-derived nanovesicle research

Early ultrastructural evidence for vesicle release by plant cells predates the therapeutic literature. In 1967, Halperin and Jensen described multivesicular bodies fusing with the plasma membrane and releasing their contents into the cell-wall space in carrot suspension cultures (Halperin and Jensen, 1967). These observations provided an early structural basis for investigating plant extracellular vesicles, whereas the therapeutic applications of edible plant-derived preparations were explored decades later.

The early therapeutic literature established two connected directions in 2013: grape-derived ELNs regulated Lgr5^+^ intestinal stem-cell responses and organoid formation (Ju et al., 2013), while Wang et al. reassembled grapefruit-derived lipids into engineered nanovectors for therapeutic delivery (Wang et al., 2013). These reconstructed carriers should be distinguished from native vesicles with demonstrated extracellular origin. In 2014, grapefruit nanovesicles combined intrinsic intestinal macrophage targeting, HO-1 induction, anti-inflammatory effects, and methotrexate delivery (Wang et al., 2014). Subsequent ginger studies in 2016 demonstrated epithelial protection (Zhang et al., 2016), while broccoli studies in 2017 established DC-associated immune regulation (Deng et al., 2017).

Teng et al. demonstrated uptake of plant-derived ELNs by gut microorganisms and RNA-mediated changes in bacterial gene expression and tryptophan metabolism in 2018 (Teng et al., 2018). Together, these studies extended edible plant nanovesicle research from epithelial and immune-cell responses to engineered delivery and microbiota–host communication. Table 1 summarizes these representative therapeutic milestones, including publication years, journals, principal findings, and scientific significance.

#### 2.1.2 Definition and terminology

Extracellular vesicles (EVs) are generally defined as non-replicating particles released by cells and enclosed by a lipid bilayer. MISEV2023 emphasizes that biogenesis-specific terms such as ‘exosome’ should be used cautiously in the absence of definitive evidence for the corresponding biogenetic pathway, a principle that is particularly relevant to plant-derived nanoparticles whose origins and biogenesis remain incompletely resolved (Welsh et al., 2024).

A critical distinction concerns *bona fide* plant extracellular vesicles (PEVs, also termed PDEVs) and exosome-like nanoparticles (ELNs). PEVs/PDEVs are non-replicating, lipid-bilayer-enclosed particles released by plant cells into the extracellular compartment. Many preparations described as ELNs, plant-derived nanoparticles (PDNPs), or related particles instead originate from juicing, homogenization, or mechanical disruption of plant tissues. Their isolation does not establish extracellular biogenesis; they may contain extracellular and intracellular vesicles, membrane fragments, lipid–protein complexes, and other nanoscale constituents (Alsaid et al., 2026; Welsh et al., 2024; Lian et al., 2022). ELNs/PDNPs and *bona fide* PEVs are therefore not interchangeable. Size and an exosome-like morphology do not demonstrate an endosomal origin, and the presence or proportion of genuine extracellular vesicles must be established for each preparation.

Throughout this review, PDEVs and PEVs denote extracellularly released vesicles when that origin is supported; neither acronym is used as an umbrella for all plant nanoparticles. The descriptive phrase “plant-derived vesicular preparations” refers collectively to the related preparations reviewed here without assigning them a shared biogenesis. Original study designations, including ELNs, GDNPs, GDNs, EVLPs, and study-specific EV labels, are retained for individual publications. Such labels alone do not establish extracellular or endosomal origin. Plant sources are specified where abbreviations overlap. Reconstructed lipid/membrane carriers and hybrid formulations are identified separately from intact native preparations (Welsh et al., 2024; Lian et al., 2022). Much foundational work predates current EV guidance, and improved terminology does not diminish its biological or therapeutic contribution.

Plant sources currently investigated in intestinal studies include grape, grapefruit, ginger, broccoli, turmeric, garlic, ginseng, *Portulaca oleracea*, *Aloe vera*, *Polygonatum sibiricum*, *Coptis chinensis*, *Andrographis paniculata*, and *Sophora japonica* (Li et al., 2025b; Zeng et al., 2024). Particles derived from different species and tissues vary substantially in their lipid, small-RNA, protein, and phytochemical compositions; these differences may in turn influence stability, target-cell uptake, and biological activity (Zeng et al., 2024; Shu et al., 2025). Plant variety, maturation stage, growth environment, harvesting, and storage conditions may further introduce batch-to-batch variability. At present, there are no universally accepted molecular markers or harmonized criteria for product identity and quality assessment across diverse plant-derived vesicular preparations (Na et al., 2026).

### 2.2 Comparison with animal‐derived EV platforms

Compared with animal-derived EVs, particularly mammalian cell-derived systems, plant preparations have distinctive lipid and phytochemical profiles. Grape ELNs are enriched in phosphatidic acid and phosphatidylethanolamine, ginger nanoparticles contain phosphatidic acid and galactolipids, and grapefruit nanovesicles have a different phospholipid profile (Ju et al., 2013; Wang et al., 2014; Zhang et al., 2016). These examples illustrate source-dependent composition, not a universal plant membrane signature. Edible biomass can offer abundant renewable feedstock and promising particle yields, while several preparations have shown gastrointestinal compatibility in preclinical studies (Fang and Liu, 2022; Wang et al., 2014; Zhang et al., 2016; Lian et al., 2022).

Plant sourcing reduces concern about mammalian donor-derived pathogens and some donor-cell immune components, but neither lower immunogenicity nor complete freedom from pathogen risk has been established across all products. Allergens, microbial contamination, pesticides, and route-dependent exposure remain relevant. Potential scalability also depends on purification recovery, defined product identity, and reproducible potency; high biomass availability is not equivalent to high yield of verified extracellular vesicles (Welsh et al., 2024; Lian et al., 2022; Shu et al., 2025; Na et al., 2026). Table 2 compares these platforms and explicitly separates potential advantages from limitations and the absence of standardized head-to-head evidence.

**TABLE 2:** Comparison of plant-derived preparations and animal-derived extracellular vesicles.

Feature	Plant-derived preparations	Animal-derived EVs	Interpretation/limitation
Source and identity	Extracellular fluids or processed edible/medicinal tissues; many therapeutic preparations are ELNs/PDNPs	Animal cells, tissues, or biofluids; identity depends on source and isolation	Biomass-derived particle yield cannot be equated with yield of verified extracellular vesicles (Welsh et al., 2024; Lian et al., 2022)
Membrane and cargo	Source-dependent phospholipids/galactolipids, RNAs, proteins, and phytochemicals	Source-dependent lipids, RNAs, and proteins; many mammalian EVs carry donor-cell membrane proteins	Plant lipid profiles vary; no single composition defines all preparations (Ju et al., 2013; Wang et al., 2014; Zhang et al., 2016; Welsh et al., 2024; Lian et al., 2022)
Oral gastrointestinal use	Several preparations show stability or intestinal activity in preclinical studies	Delivery behavior varies; oral formulations may require protection	Compatibility must be demonstrated for each product and cargo (Fang and Liu, 2022; Wang et al., 2014; Zhang et al., 2016; Huang et al., 2026)
Immunological considerations	Favorable preclinical tolerability in selected systems; plant allergens and route-dependent exposure remain relevant	Immunogenicity depends on cell source, cargo, purification, and exposure	Lower plant-platform immunogenicity is a potential advantage, not an established universal comparison (Welsh et al., 2024; Lian et al., 2022; Na et al., 2026)
Pathogen-related concerns	Reduced concern about mammalian donor-derived pathogens; microbial contamination still requires control	Source-cell and donor-associated adventitious agents require control	Plant origin does not establish pathogen-free pharmaceutical quality (Welsh et al., 2024; Lian et al., 2022; Na et al., 2026)
Feedstock and scalability	Abundant renewable biomass and potentially high particle recovery	Controlled cell culture/bioreactors or donor/biofluid sourcing	Clinical-grade scalability requires purity, batch consistency, and reproducible potency in both platforms (Lian et al., 2022; Shu et al., 2025; Wang et al., 2022)
Biogenesis and heterogeneity	Extracellular origin often unresolved after tissue disruption	Many mammalian EV pathways are better characterized; isolated populations remain heterogeneous	Define isolation, identity, and co-isolates rather than assume equivalence (Alsaid et al., 2026; Welsh et al., 2024; Lian et al., 2022)
Translation and quality control	Predominantly preclinical intestinal evidence; product identity is a central obstacle	Broader therapeutic development experience; potency and manufacturing still challenging	No standardized head-to-head superiority is established (Welsh et al., 2024; Na et al., 2026; Li and Toh, 2026)

The plant column covers related vesicular/nanoparticle preparations and does not equate tissue-derived ELNs/PDNPs with extracellularly released vesicles. Animal-derived EVs here primarily refer to mammalian systems commonly used in therapeutic research. Statements are general platform tendencies, not proof of superiority. ELNs, exosome-like nanoparticles; PDNPs, plant-derived nanoparticles.

### 2.3 Isolation and characterization

The most frequently used methods for enriching plant-derived nanovesicles include differential centrifugation and ultracentrifugation, with some studies using density-gradient centrifugation to achieve higher purity. Size-exclusion chromatography, membrane filtration and tangential-flow filtration (TFF) have also been gaining momentum, especially since TFF is scalable and compatible with continuous processing (Urzì et al., 2021; Liu Z. et al., 2025).

Routine characterization uses transmission electron microscopy for morphology, nanoparticle tracking analysis (NTA) or dynamic light scattering (DLS) for size distributions, and zeta-potential measurements for surface charge. These measurements alone cannot establish preparation identity or purity. Complementary separation methods, membrane-integrity assessment, and proteomic, lipidomic, small-RNA, or metabolomic analyses are needed to characterize vesicular and non-vesicular constituents (Liu Z. et al., 2025; Zhang et al., 2025).

Isolation workflows balance purity, recovery, membrane integrity, reproducibility, and scalability. Because processing can alter constituent proportions and biological potency, isolation methods should be treated as determinants of product identity and function when comparing studies (Yu et al., 2026).

### 2.4 Bioactive cargo and functional membrane components

Plant-derived vesicular preparations can contain RNAs, proteins, and phytochemicals associated with lipid membranes. Their composition, membrane integrity, and non-vesicular constituents must be considered when attributing biological activity (Welsh et al., 2024; Huang et al., 2025). Ginger-derived nanoparticles (GDNPs 2) contain phosphatidic acid and galactolipids, small RNAs, 6-gingerol and 6-shogaol; uptake and anti-inflammatory activity were investigated in RAW264.7 macrophages, while epithelial effects were studied in Colon-26 and Caco-2BBE systems and in mice (Zhang et al., 2016). These findings do not identify the proportional contribution of each constituent. Garlic-derived exosome-like nanovesicles, termed GENs in the original study, provide more targeted evidence: han-miR3630-5p was linked to the TLR4 3′ untranslated region and suppression of TLR4/MyD88/NF-κB signaling in a study of DSS-induced mouse colitis (Zhu Z. et al., 2023).

Plant nanovesicle research has progressed in recent years beyond compositional profiling to causal validation of functional cargo. The extracellular vesicle-like particles (EVLPs) derived from *Prunus mume* disrupt the NEK7–NLRP3 interaction and inhibit NLRP3 inflammasome activation; moreover, RNA degradation, small-RNA sequencing and experiments with miRNA mimics and inhibitors support the key role of miR159 as one of the functional cargoes (Lv et al., 2025). In ELNs from *P. sibiricum*, miR166u has been reported to target CD11c and is associated with inhibition of the TLR4/AKT/NF-κB pathway (Wei et al., 2025). The ELNs derived from *C. chinensis* provide mechanistic evidence linking miR-5106, Slc39a2, zinc homeostasis, NET formation, reduced IEC pyroptosis, and increased IEC/ISC proliferation (Yang Y. et al., 2025). Notably, however, this study was performed using intraperitoneal administration; the main contribution of this study is thus mechanistic validation and not direct evidence of effective oral delivery.

In addition to encapsulated cargo, the plant nanovesicle membrane components could also play a role in targeting and functional regulation. Grape-derived GELN lipids contribute to the response of Lgr5^+^ ISCs (Ju et al., 2013), while membrane lipids of ginger-derived ELNs are involved in preferential uptake by Lactobacillaceae (Teng et al., 2018). Plant nanovesicle function is therefore best understood as an integrated ‘membrane properties–bioactive cargo–receptor targets’ framework–where membrane lipids and surface components affect cargo stability, uptake by host cells or microorganisms, and tissue distribution, while RNAs and plant-derived small molecules control molecular targets and signaling pathways (Hao et al., 2024; Wu et al., 2024). In the future, functional attribution should increasingly rely on causal approaches such as cargo depletion, receptor blockade, and rescue experiments, rather than on association alone based on omics data.

Membrane composition and an uptake mechanism should not be conflated. Lipid profiling identifies constituents, whereas inhibitor, competition, depletion, or lipid-reconstitution experiments can provide functional evidence for proposed uptake mechanisms. For example, the grapefruit study supported macropinocytosis and clathrin-dependent entry in RAW264.7 cells using inhibitors (Wang et al., 2014), and bayberry galactose-competition experiments supported a carbohydrate-associated process without establishing a unique receptor (Wang M. et al., 2026). Table 3 separates membrane characteristics, intestinal targets, proposed uptake routes, and the actual form of uptake evidence.

**TABLE 3:** Membrane characteristics and intestinal uptake evidence of representative plant preparations.

Plant/designation	Reported membrane feature	Target and evidence system	Uptake mechanism and support	Uptake measurement/limitation	Refs.
Grape GELNs	PA 53.2%; PE 26.1% of the reported lipid profile	Lgr5^+^ cells; oral mouse exposure and crypt/ISC organoid cultures	Reconstructed source lipids supported ISC targeting; a specific surface receptor was not established	Fluorescence and cellular/ISC responses; no common absolute delivery-efficiency measure	(Ju et al., 2013)
Grapefruit GDNs	PE/PC-rich profile; membrane determinants not individually assigned to uptake	RAW264.7 cells; intestinal F4/80^+^ macrophages; DSS-colitis mice	Amiloride/chlorpromazine inhibition supported macropinocytosis and clathrin-dependent entry	∼30% of colonic F4/80^+^ cells were label-positive in a specified DSS protocol. In RAW264.7 cells, amiloride and chlorpromazine reduced uptake by 45.48% ± 15.64% and 58.98% ± 15.21%, respectively (GDN exposure: 2 μg/mL, 3 h). These are relative reductions, not absolute delivery efficiencies	(Wang et al., 2014)
Ginger GDNPs 2	PA and galactolipids DGDG/MGDG enriched across GDNP fractions	Epithelial and RAW264.7 cell systems; orally treated mice	Internalization demonstrated; lipid composition alone does not establish a shared endocytic route	Cell-associated fluorescence/flow cytometry within the original experiment; no standardized cross-study efficiency	(Zhang et al., 2016)
Ginger ELNs	PA-enriched lipids; uptake dependence examined by lipid perturbation	Lactobacillaceae, including L. rhamnosus GG; bacterial cultures and mouse gut	Lipid-dependent preferential microbial uptake; RNA cargo affected bacterial genes and metabolites	Bacterial labeling and functional assays; bacterial and mammalian-cell uptake denominators differ	(Teng et al., 2018)
Broccoli BDNs	Lipid fraction with sulforaphane-associated activity	Bone marrow-derived/intestinal DC systems; mouse colitis	DC targeting demonstrated; lipid/cargo depletion and AMPK perturbation address bioactivity rather than identify an uptake receptor	Labeled-particle uptake and DC-transfer effects; a universal numerical uptake efficiency was not established	(Deng et al., 2017)
Bayberry BELNs	MGDG/DGDG and a galactose-associated membrane signature	RAW264.7 macrophages *in vitro*; oral DSS-colitis mice	Reduced uptake with free-galactose competition supports a carbohydrate-associated process; receptor specificity remains unresolved	PKH26 microscopy and flow cytometry (8 μg/mL, 5 h); within-study competition, without a common efficiency scale	(Wang et al., 2026a)

Lipid percentages describe the reported analytical profiles and are not uptake percentages. The grapefruit numerical values are protocol-specific positive-cell or inhibitor-effect measurements, not percentages of the administered dose delivered intact. Label-based uptake can be confounded by free dye or membrane exchange. No between-study efficiency ranking is intended. PA, phosphatidic acid; PE, phosphatidylethanolamine; PC, phosphatidylcholine; DGDG, digalactosyldiacylglycerol; MGDG, monogalactosyldiacylglycerol.

### 2.5 Gastrointestinal stability, mucosal retention, and cellular uptake

The intestinal activity of orally administered plant-derived vesicular preparations depends on the stability and local availability of their bioactive constituents, as well as their interactions with target host cells or gut microorganisms. Gastric acidity, digestive enzymes and bile salts are the first hurdles for plant-derived vesicular preparations to overcome (Fang and Liu, 2022). Their relative stability may be attributed to the physical protection of the membrane, which could protect encapsulated phytochemicals and RNAs, and to the properties of specific membrane-lipid compositions; these lipids could help maintain membrane integrity and colloidal stability under acidic and digestive conditions (Li H. et al., 2026; Huang et al., 2026). Depending on the preparation and its mechanism of action, interactions with the intestinal mucus layer, access to target sites, cellular or microbial uptake, and functional cargo release may contribute to biological activity. Different nanoparticles have been reported to be gastrointestinally stable or enriched in the colon after preparation from grapefruit (Wang et al., 2014), ginger (Zhang et al., 2016), *P. oleracea* (Zhu M. Z. et al., 2023), *P. sibiricum* (Wei et al., 2025), and *A. paniculata* (Zhu et al., 2026).

Plant-derived vesicular preparations from different sources also show varying cellular/microbial uptake in the intestine. Grapefruit-derived GDNs are preferentially taken up by intestinal macrophages (Wang et al., 2014), ginger-derived GDNPs enter IECs and macrophages (Zhang et al., 2016), while grape-derived GELNs interact with Lgr5^+^ ISCs (Ju et al., 2013). Some plant-derived vesicular preparations are also able to be internalized by certain gut microbes, as a potential platform for delivery of functional RNAs and manipulation of microbial behavior (Wang et al., 2024). In addition to these inherent uptake mechanisms, the accumulation and uptake of plant-derived vesicular preparations in diseased intestinal mucosa and targeted cell types can be improved by engineering strategies.

It is important to note that any fluorescence detected in the tissue following oral delivery of plant-derived vesicular preparations should not necessarily be taken as evidence that the vesicles and/or payload have survived to reach the target location (Lázaro-Ibáñez et al., 2021). Delivery assessment should distinguish intact-particle arrival, cargo co-delivery, and functional target-cell responses from tissue-associated fluorescence (Zhang Q. et al., 2026).

Reported uptake metrics are not directly interchangeable: they may represent the fraction of administered particles internalized, the proportion of cells positive for a particle-associated label, or fluorescence intensity normalized to a control (Wang et al., 2014). These are different measurements. Consequently, Table 3 includes verified within-study numerical observations where available, but does not rank preparations using an artificial common efficiency scale. Lipophilic-label uptake remains subject to the integrity and free-label limitations described above (Welsh et al., 2024; Lázaro-Ibáñez et al., 2021).

## 3 Regulation of intestinal inflammation and the immune microenvironment by plant-derived vesicular preparations

### 3.1 Innate immune regulation

Current studies of plant-vesicle-mediated regulation of intestinal innate immunity have focused primarily on macrophages, inflammasomes, and neutrophil-associated injury.

Macrophages are among the best-characterized targets of plant-derived vesicular preparations. The foundational grapefruit study used RAW264.7 macrophages *in vitro*, isolated intestinal lamina propria macrophages *ex vivo*, and oral treatment in C57BL/6J mice with DSS-induced colitis. Grapefruit-derived nanovesicles (GDNs) induced HO-1 and reduced IL-1β and TNF-α, linking cellular uptake to intestinal immune modulation (Wang et al., 2014). *Lycium barbarum* lipid-derived nanoparticles (LBLNs), reconstituted from wolfberry fat-soluble components, should be distinguished from intact native vesicular preparations. In RAW264.7 macrophages, LBLNs showed cellular uptake, reduced TNF-α and IL-12 secretion, and increased IL-10 expression. Oral administration promoted accumulation in inflamed colonic tissue and ameliorated experimental mouse colitis (Zu et al., 2020). Ginger-derived GDNPs likewise showed macrophage-associated anti-inflammatory activity (Zhang et al., 2016).

Ginseng-derived exosome-like nanoparticles (GENs) were evaluated in Caco-2 cells and LPS-stimulated RAW264.7 macrophages, and administered by oral gavage in DSS-induced acute colitis in male BALB/c mice, with suppression of inflammatory cytokines (Kim et al., 2023). Turmeric-derived nanoparticles (TDNPs 2) were examined in RAW264.7 macrophages, Colon-26 cells, and Caco-2BBE cells, and administered orally in DSS-induced colitis and post-DSS recovery models in female FVB/NJ mice, with anti-inflammatory and recovery-associated effects (Liu et al., 2022). In LPS-stimulated RAW264.7 cells and DSS-induced mouse colitis, *A. paniculata* ELNs shifted macrophage-associated phenotypes toward M2 features alongside PI3K-AKT/JAK-STAT-related changes (Zhu et al., 2026). Bayberry ELNs were examined in RAW264.7 cells and orally treated DSS-colitis mice; free-galactose competition supported a galactose-associated uptake process in the cultured macrophages (Wang M. et al., 2026). *Magnolia biondii* decoction-derived exosome-like nanoparticles (MFELNs) were evaluated in LPS-stimulated RAW264.7 macrophages and administered by oral gavage in male C57BL/6 mice with DSS-induced colitis, with reductions in inflammatory and oxidative-stress readouts (Yue et al., 2026).

Tea-derived preparations, termed TEVs in the original study, showed time-dependent macrophage uptake and promoted M2-associated polarization *in vitro*, with partly RNA-dependent protection in DSS-induced mouse colitis. TEV-derived osa-miR166d-5p and gma-miR396a-3p targeted AKT1 and IKBKB, respectively, reducing NF-κB-associated signaling (Luo et al., 2025). These results support recurring macrophage effects, but do not establish equivalent uptake routes or potency across sources.

Inflammasome studies provide increasingly specific mechanistic evidence. *Houttuynia cordata* ELNs were evaluated using RAW264.7, NCM460, SW480, and HT29 uptake systems and oral treatment of DSS-colitis mice, including NLRP3-deficient animals; the knockout comparison supported involvement of NLRP3-associated signaling (Li J. H. et al., 2024). *Prunus mume*-derived EVLPs were investigated in bone marrow-derived macrophages (BMDMs), PMA-differentiated THP-1 cells, and DSS- and TNBS-induced mouse colitis. RNA perturbation and miR159 mimic/inhibitor experiments supported disruption of NEK7-NLRP3 interaction; macrophage depletion further linked protection to this cell population (Lv et al., 2025). *Sophora japonica*-derived preparations, termed Flos Sophorae immaturus exosome-like nanovesicles (FSIEVs) in the original study, reduced NLRP3-associated inflammation and oxidative stress in DSS-induced mouse colitis (Wu et al., 2026). Genetic or cargo perturbations strengthen these mechanisms, but effects on a pathway should still be distinguished from direct proof of complete mucosal restoration.

Aberrant neutrophil activation and NET formation connect immune-mediated injury to epithelial recovery. *Coptis chinensis* ELNs were studied in DSS-induced colitis mice, primary neutrophils isolated from mouse bone marrow and spleen, and NET-exposed mouse IECs. Delivery of miR-5106 reduced Slc39a2 expression and restored neutrophil zinc homeostasis, with reduced NET formation and epithelial pyroptosis and increased IEC/ISC-associated proliferation (Yang Y. et al., 2025). Importantly, the particles were administered intraperitoneally *in vivo*; these data support an immune-epithelial mechanism but cannot establish effective oral delivery. Moreover, increased Lgr5/Ki67-associated readouts do not by themselves prove durable stem-cell-mediated regeneration.

The study of plant-vesicle-mediated innate immunity is therefore progressing from descriptions of cytokine changes toward mechanistic evidence linking ‘specific immune cells–functional cargo–molecular targets–tissue outcomes’. Although a wealth of evidence exists on macrophage polarization, the NLRP3 inflammasome, and NETs, the relative contribution of each of these immune pathways to final mucosal repair is not well defined. Future studies should assess inflammatory mediators, immune-cell states, epithelial cell death, crypt architecture, and ISC activity to determine whether immune modulation is accompanied by tissue repair.

### 3.2 Mucosal and adaptive immune regulation

Evidence that plant-derived vesicular preparations regulate the classic adaptive immune-cell populations is comparatively limited relative to that for innate immunity. Current research has concentrated mainly on dendritic cells, T-cell-associated responses and mucosal homeostatic pathways like AhR-IL-22 signaling, all of which indicate modulation of the mucosal immune environment.

Broccoli-derived nanoparticles represent a foundational demonstration of dendritic-cell targeting. Deng et al. used bone marrow-derived DC cultures, DSS-induced colitis, naive CD4^+^ T-cell-transfer colitis in Rag1-deficient mice, anti-CD40-induced colitis, and adoptive DC transfer. Oral BDNs promoted an AMPK-associated tolerogenic phenotype; transfer of DCs exposed to total BDN lipids, but not sulforaphane-depleted fractions, supported a contribution of sulforaphane. Comparison of AMPK-competent and AMPK-deficient DCs further connected this cargo-associated effect to AMPK (Deng et al., 2017). Thus, the study supplied cellular and *in vivo* mechanistic evidence, rather than only an association between reduced cytokines and improved disease.

*Portulaca oleracea*-derived ELNs were evaluated by oral administration in DSS-induced and IL-10-deficient mouse colitis, with intestinal T-cell phenotyping. Expansion of CD4^+^CD8^+^ double-positive T cells and AhR-associated responses accompanied protection (Zhu M. Z. et al., 2023). Mulberry-bark ELNs were studied in DSS-colitis mice, including epithelial-specific COPS8 knockout animals; loss of epithelial COPS8 attenuated the antimicrobial-peptide and protective responses downstream of AhR (Sriwastva et al., 2022). These experiments distinguish intestinal epithelial signaling from direct immune-cell targeting. *Sophora japonica*-derived ELNs provide a further microbiota-associated AhR example, involving tryptophan metabolism and improved mucosal inflammation (Wu et al., 2026).

*Robinia pseudoacacia* flower ELNs (RFELNs) were administered by oral gavage to male C57BL/6J mice with DSS-induced colitis, with colonic ILC3/NCR+ ILC3 phenotyping and MNK-3 cells used as an *in vitro* ILC3-like system. AhR-associated signaling and IL-22 induction accompanied improvements in goblet-cell and junctional readouts and reduced FITC-dextran permeability (Shen et al., 2026). These findings support immune–epithelial communication, although the MNK-3 model does not establish equivalent responses in primary human ILC3s.

Current evidence suggests two broad modes of plant-vesicle-mediated mucosal immune regulation: direct intracellular signaling in immune cells such as DCs, and modulation of immune–epithelial crosstalk through mucosal homeostatic pathways involving AhR, IL-22, and antimicrobial defense. Direct evidence for regulation of Treg, Th17, B-cell, and other adaptive immune subsets, however, remains sparse. Plant-derived vesicular preparations should therefore not yet be generalized as broad-spectrum regulators of adaptive immunity.

### 3.3 Linking inflammatory control to mucosal repair

Inflammatory control can reduce epithelial injury and improve the regenerative environment, but does not establish complete mucosal repair (Ho et al., 2020; Holmberg et al., 2018). Evidence should distinguish disease amelioration, inflammatory control, structural repair, regenerative activity, and functional recovery. Cytokine reduction primarily supports anti-inflammatory activity; junctional and mucus-associated changes, ISC responses, and permeability measurements address distinct repair outcomes. Combining these endpoints within the same model can clarify whether immune modulation is accompanied by tissue and barrier recovery.

Mechanically dynamic and immuno-instructive biomaterials provide a complementary perspective on regenerative microenvironments (Puri et al., 2026), motivating studies of carrier–matrix effects on intestinal immunity and epithelial function. This is a design analogy rather than direct evidence of a plant nanoparticle mechanism.

## 4 Evidence for intestinal barrier repair and epithelial regeneration

### 4.1 Epithelial protection, proliferation, and stem cell-driven regeneration

Epithelial survival, proliferation, and stem-cell-associated responses represent distinct contributions to mucosal repair. The foundational ginger nanoparticle study combined epithelial cell assays with oral treatment of mouse colitis and colitis-associated cancer models (Zhang et al., 2016). More recently, broccoli sprout-derived preparations, termed BSDExo by the original authors, were tested in CCD 841 CoN normal colonic epithelial cells and Caco-2 monolayers. They promoted epithelial proliferation and improved LPS- or DSS-injured monolayer integrity (Yang T. et al., 2026). These are *in vitro* repair data; animal efficacy was proposed as future work and should not be inferred from the cell-based findings.

Protection and proliferation of mature IECs are fundamental mechanisms of recovery of the epithelium, and sustained tissue regeneration is reliant on ISC function. The most direct evidence for plant-vesicle-mediated regulation of ISCs comes from grape-derived exosome-like nanoparticles (GELNs). They interact with Lgr5^+^ ISCs and promote organoid formation when provided to isolated intestinal crypts or sorted Lgr5^+^ cells; GELN-derived lipids contribute to ISC targeting, and suppression of β-catenin signaling attenuates the regenerative response (Ju et al., 2013). Responses in Lgr5^+^ cells and functional organoid formation are more direct indicators of ISC involvement in epithelial regeneration when compared to increased Ki67 expression or crypt hyperplasia alone. Long-term lineage tracing and sustained functional assessment are still needed to establish whether these short-term stem-cell responses produce stable epithelial replenishment and durable tissue reconstruction.

In sum, there is some evidence that plant-derived vesicular preparations can directly promote epithelial and ISC function to aid in mucosal regeneration. However, research that includes long-term lineage tracking, ISC-specific functional manipulation and continuous evaluation of tissue regeneration is scarce.

### 4.2 Reconstruction of epithelial junctions and the mucus barrier

Several plant-derived preparations improve structural readouts of the intestinal mucosal barrier. *Aloe*-derived nanovesicles were associated with recovery of tight- and adherens-junction proteins (Choi et al., 2023), while *P. sibiricum* ELNs increased ZO-1, occludin and MUC2 and improved injured mucosal architecture (Wei et al., 2025). These findings support structural repair, but changes in individual proteins do not by themselves establish complete barrier reconstruction or functional recovery.

In high-fat-diet-aggravated colitis, Citrus-derived ELNs increased occludin and ZO-1, and improved oxidative-stress, microbial and metabolic readouts (Zhan et al., 2026). These results broaden the settings in which junctional and barrier-associated improvements have been observed. The contribution of each effect to sustained functional mucosal recovery still requires direct assessment.

### 4.3 From structural repair to functional barrier restoration

Improvement in structural markers alone does not ensure that barrier function is restored. Molecules like ZO-1, occludin, claudins and MUC2 mainly report changes in the structure of the cell junctions and the mucus layer, while measurements like transepithelial electrical resistance (TEER), FITC-dextran permeability, bacterial translocation, and other trans-barrier transport measurements provide functional evidence in a more direct fashion (Vieujean et al., 2026; Rath et al., 2023).

Some studies assess functional barrier outcomes directly. Broccoli sprout-derived BSDExo improved TEER in LPS- or DSS-injured Caco-2 monolayers, providing *in vitro* functional evidence (Yang T. et al., 2026). *Robinia pseudoacacia* flower ELNs improved junctional and goblet-cell readouts and reduced FITC-dextran permeability in mice (Shen et al., 2026). *Aloe*-derived nanovesicles were also associated with improved intestinal permeability in acute experimental colitis (Choi et al., 2023). The biological setting matters: recovery of electrical resistance in a cell monolayer and reduced permeability in an animal are complementary findings, not interchangeable levels of clinical evidence.

A remaining question is whether barrier improvements persist after treatment withdrawal or secondary injury and are accompanied by normal epithelial renewal. Integrated assessment of structural, regenerative, and functional outcomes is therefore needed to establish the depth and durability of repair (Figure 1).

**FIGURE 1:**
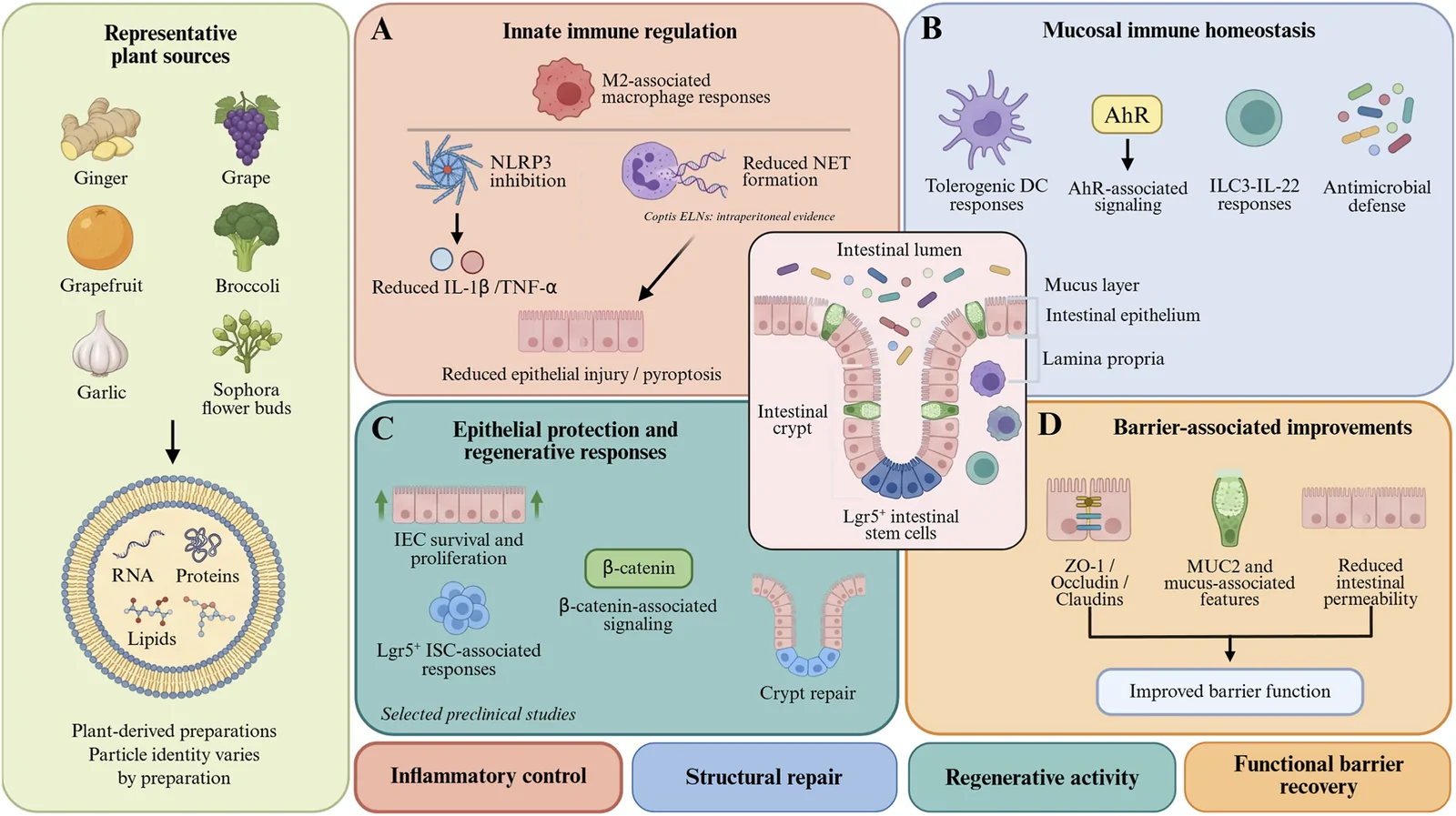
Multilevel mechanisms by which plant-derived vesicular preparations support intestinal mucosal repair. **(A)** Innate immune regulation. **(B)** Mucosal immune homeostasis. **(C)** Epithelial protection and regenerative responses. **(D)** Barrier-associated improvements. Depicted ELN/PDNP preparations should not be assumed to represent *bona fide* extracellular vesicles without biogenetic evidence. Abbreviations: ELNs, exosome-like nanoparticles; PDNPs, plant-derived nanoparticles; M2, M2‐associated macrophage phenotype; NLRP3, NOD-like receptor family pyrin domain-containing 3; NETs, neutrophil extracellular traps; IL-1β, interleukin-1 beta; TNF-α, tumor necrosis factor alpha; DC, dendritic cell; AhR, aryl hydrocarbon receptor; ILC3, group 3 innate lymphoid cell; IL-22, interleukin-22; IEC, intestinal epithelial cell; ISC, intestinal stem cell; Lgr5, leucine-rich repeat-containing G-protein-coupled receptor 5; ZO-1, zonula occludens-1; MUC2, mucin 2. Created in BioRender. liu, W. (2026) https://BioRender.com/0dspl6u.

## 5 Plant-derived preparations, gut microbiota, and mucosal repair

### 5.1 Gut microorganisms as direct targets of plant-derived preparations

Selected plant-derived preparations can be taken up by gut bacteria and alter bacterial growth, gene expression, or stress adaptation through membrane components, RNAs, or other bioactive constituents. These findings suggest that direct cross-kingdom interactions may contribute to microbiota remodeling. For example, peu-MIR2916-p3 in garlic-derived ELNs promotes the growth of *Bacteroides thetaiotaomicron*, demonstrating that plant miRNAs can directly influence specific commensal bacteria (Wang et al., 2024). Plant-derived vesicular preparations may also influence microbial adaptation to the gastrointestinal environment. Lemon-derived ELNs enhance bile tolerance and intestinal survival of *Lactobacillus rhamnosus* GG and *Streptococcus thermophilus* and alter the production of lactate and indole metabolites. Their pectin-like polysaccharides can further regulate *Lactobacillus* protein expression and increase bile tolerance through RNase P-associated, selective tRNA degradation (Lei et al., 2020; Lei et al., 2021). Together, these findings show that plant-vesicle–microbe interactions operate at multiple levels, including selective uptake, regulation of bacterial growth, reprogramming of gene expression, and adaptation to environmental stress.

### 5.2 Functional microbiota remodeling and metabolic relays

The ecological effects induced by plant-derived vesicular preparations extend beyond changes in microbial composition and can alter microbial metabolic function. The resulting microbial metabolites may serve as critical ‘metabolic relays’ that connect plant nanovesicle exposure to host mucosal responses, with current evidence centering primarily on tryptophan-derived metabolites and short-chain fatty acids.

Tryptophan-indole metabolism represents one of the most mechanistically defined routes through which plant-derived vesicular preparations mediate microbiota-host crosstalk. The foundational microbiota study by Teng et al. showed that ginger-derived ELNs influence host AhR-associated mucosal homeostasis by promoting the production of indole-3-carboxaldehyde (Teng et al., 2018). *Sophora japonica*-derived nanovesicles (FSIEVs) increased *Lactobacillus* abundance and indole-3-acetic acid (IAA) production in DSS-induced mouse colitis. Antibiotic treatment, fecal microbiota transplantation (FMT), L. paracasei supplementation, IAA intervention, and AhR antagonism supported a microbiota–metabolite–host receptor pathway, while organoid experiments supported IAA-mediated protection (Wu et al., 2026). Similarly, garlic-derived ELNs increase *Lactobacillus reuteri* in weaned piglets, together with changes in microbial metabolites such as indole-3-propionic acid and improved intestinal homeostasis (Liu X. et al., 2025). Although different plant-derived vesicular preparations may affect distinct indole derivatives, these studies converge on a common principle: remodeling of microbial tryptophan metabolism converts changes in microbiota function into host mucosal responses.

Recent work further suggests that plant-vesicle-mediated metabolic regulation extends beyond the tryptophan–indole axis. *Sargassum fusiforme*-derived ELNs remodel the gut microbiota, increase taxa including *Muribaculum intestinale*, and promote short-chain fatty acid production, in parallel with improvements in intestinal barrier function and inflammatory status (Chang et al., 2026). Perilla-derived nanovesicles similarly enrich bacteria associated with carbohydrate fermentation and restore cecal short-chain fatty acid levels, while FMT experiments further support a causal contribution of microbiota remodeling to their protective effects (Yang Z. et al., 2026).

Plant-vesicle-microbiota studies should therefore progress beyond descriptive changes in community composition and metabolite profiles toward causal validation using FMT, mono-colonization, metabolite intervention, or host-receptor blockade. The field is beginning to move from descriptive observations of ‘microbiota changes’ toward causal pathways linking specific microorganisms, metabolites, and host responses. Plant nanovesicle efficacy may also depend on the host’s baseline microbial ecosystem. Future studies using germ-free animals, defined microbial communities, and patient-derived microbiota models should help clarify this context dependence and its translational relevance. Across Sections 3-5, the depth of evidence differs markedly among plant-derived vesicular preparations with respect to inflammatory control, mucosal immune regulation, structural barrier repair, epithelial regeneration, and microbiota-host interactions; representative studies and their corresponding levels of mucosal-repair evidence are summarized in Table 4. Taken together, these data indicate that plant-derived vesicular preparations not only directly reshape gut microorganisms but also transmit microbial signals to host immunity and the epithelial barrier through changes in microbial function and metabolites, thereby establishing a plant-vesicle-microbiota-host mucosal repair axis (Figure 2).

**TABLE 4:** Representative studies of plant-derived vesicular preparations in intestinal mucosal repair.

Plant/reported preparation	Main target	Experimental system/evidence setting	Delivery/exposure	Mechanism and outcome	Repair evidence category	Refs.
Grape/GELNs	Lgr5^+^ ISCs	DSS-colitis mice; Lgr5 reporter mice; isolated crypts and sorted Lgr5^+^ organoid cultures (*in vivo* + ex vivo-derived culture)	Oral	Source lipids and β-catenin-associated responses; increased ISC activity and organoid formation	Regenerative activity	(Ju et al., 2013)
Ginger/GDNPs 2	IECs; macrophages	RAW264.7, Colon-26 and Caco-2BBE assays; acute/chronic mouse colitis and colitis-associated cancer (*in vitro* + *in vivo*)	Oral	Multicomponent cargo; reduced inflammation and increased epithelial survival/proliferation	Inflammation control + epithelial repair	(Zhang et al., 2016)
Grapefruit/GDNs	Intestinal macrophages	RAW264.7; isolated lamina propria macrophages; C57BL/6J DSS-colitis mice (*in vitro* + *ex vivo* + *in vivo*)	Oral	Preferential uptake; HO-1 induction; reduced IL-1β/TNF-α and colitis	Inflammation control	(Wang et al., 2014)
Broccoli/BDNs	DCs	Bone marrow-derived DCs; DSS, naive CD4^+^ T-cell-transfer (Rag1^−/−^), and anti-CD40 colitis; DC transfer (*in vitro* + *in vivo*)	Oral particles; adoptive DC transfer in mechanistic experiments	Sulforaphane-associated AMPK activation and tolerogenic DC responses	Immune regulation	(Deng et al., 2017)
*Portulaca oleracea*/ELNs	T cells; microbiota	DSS-induced and IL-10-deficient mouse colitis; intestinal CD4^+^CD8^+^ T-cell phenotyping (*in vivo*)	Oral	Microbiota/AhR-associated responses; double-positive T-cell expansion	Immune regulation	(Zhu et al., 2023b)
*Andrographis paniculata*/ELNs	Macrophages	LPS-stimulated RAW264.7 cells; DSS-colitis mice and colonic macrophage phenotyping (*in vitro* + *in vivo*)	Oral	M1/M2-associated shifts; PI3K-AKT/JAK-STAT-related signaling; barrier/microbiota changes	Inflammation control + barrier support	(Zhu et al., 2026)
Garlic/GENs	Immune-epithelial pathways	DSS-induced mouse colitis; molecular cargo/target analyses (*in vivo* with mechanistic assays)	Oral pretreatment	han-miR3630-5p–TLR4/MyD88/NF-κB; reduced inflammatory injury	Inflammation control	(Zhu et al., 2023a)
Garlic/ELNs (original title uses exosomes)	B. thetaiotaomicron	Bacterial growth assays and DSS-colitis mice (*in vitro* + *in vivo*)	Oral	peu-MIR2916-p3; promotion of an anti-colitic commensal	Microbiota-mediated repair support	(Wang et al., 2024)
Garlic/ELNs	L. reuteri-associated ecosystem	Stressed/weaned piglets; intestinal tissue, microbiota and metabolite analyses (*in vivo*)	Oral supplement	L. reuteri enrichment and IPA-associated metabolic changes	Microbiota-metabolite-host support	(Liu et al., 2025b)
*Prunus mume*/EVLPs	Macrophage NLRP3	BMDMs; PMA-differentiated THP-1; C57BL/6 DSS and BALB/c TNBS colitis; macrophage depletion (*in vitro* + *in vivo*)	Oral	miR159; NEK7-NLRP3 disruption; reduced inflammasome activation	Inflammation control with mechanistic evidence	(Lv et al., 2025)
*Polygonatum sibiricum*/ELNs	Barrier/immune pathways	DSS-induced mouse colitis; barrier, microbiota and molecular target analyses (*in vivo* with mechanistic assays)	Oral	miR166u-CD11c/TLR4-AKT; increased ZO-1, occludin and MUC2	Structural repair	(Wei et al., 2025)
*Coptis chinensis*/ELNs	Neutrophils; IECs; ISCs	Primary mouse marrow/splenic neutrophils; NET-exposed mouse IECs; DSS-colitis mice (*in vitro* + *in vivo*)	Intraperitoneal particles	miR-5106–Slc39a2–zinc/NET axis; reduced IEC pyroptosis; proliferation-associated responses	Inflammation control + regenerative markers	(Yang et al., 2025b)
*Houttuynia cordata*/ELNs	NLRP3-associated pathways	RAW264.7/NCM460/SW480/HT29 uptake assays; wild-type and NLRP3-deficient DSS-colitis mice (*in vitro* + *in vivo*)	Oral	NLRP3-dependent protection; immune and microbiota changes	Inflammation control with causal support	(Li et al., 2024a)
Mulberry bark/MBELNs	IECs	DSS-colitis mice including epithelial-specific COPS8 knockout (*in vivo*)	Oral	AhR-COPS8; increased antimicrobial defense	Mucosal immune/antimicrobial support	(Sriwastva et al., 2022)
*Flos Sophorae Immaturus*/FSIEVs (original designation)	Microbiota; IECs	DSS-colitis mice; microbiota depletion, FMT and L. paracasei intervention; DSS-injured mouse crypt-derived organoids treated with IAA (*in vivo* + tissue-derived culture)	Oral FSIEVs in mice; IAA exposure in organoid cultures	Microbiota-dependent tryptophan/IAA–AhR relay; reduced inflammation and oxidative stress; IAA-associated organoid protection	Microbiota-host immune regulation	(Wu et al., 2026)
*Robinia pseudoacacia* flowers/ELNs	ILC3; IECs	DSS-induced colitis in male C57BL/6J mice; colonic ILC3/NCR+ ILC3 phenotyping; MNK-3 cells (*in vivo* + *in vitro*)	Oral gavage	AhR/IL-22; junction/mucus improvement and reduced FITC-dextran permeability	Functional barrier recovery in mice	(Shen et al., 2026)
Aloe/Aloe-derived nanovesicles: VNVs, ANVs, and SNVs	IECs	DSS-induced acute mouse colitis; junctional-protein and intestinal-permeability assessment (*in vivo*)	*In vivo* particle administration	Tight/adherens-junction recovery and improved permeability	Structural + functional repair	(Choi et al., 2023)
Broccoli sprouts/BSDExo	Colonic epithelial cells	CCD 841 CoN proliferation; LPS-/DSS-injured Caco-2 monolayers (*in vitro*)	Cell-culture exposure	Increased epithelial proliferation and TEER; animal efficacy not established	Functional barrier recovery *in vitro*	(Yang et al., 2026a)
Citrus/ELNs	IECs; microbiota	High-fat-diet-aggravated DSS-induced mouse colitis; tissue, microbial and metabolic analyses (*in vivo*)	Dietary intervention	Increased occludin/ZO-1 and improved oxidative/metabolic readouts	Structural repair	(Zhan et al., 2026)
Lemon/ELNs	Gut bacteria	L. rhamnosus GG/S. thermophilus cultures; *C. difficile* infection in mice (*in vitro* + *in vivo* across cited studies)	Oral probiotic/ELN-related regimens	Pectin-associated RNase P/tRNA regulation; improved bile tolerance and probiotic function	Microbiota functional remodeling	(Lei et al., 2020; Lei et al., 2021)
*Perilla frutescens*/nanovesicles	Gut microbiota; immune milieu	Experimental mouse colitis and FMT (*in vivo*)	Oral	Microbiota remodeling and SCFA recovery; FMT-supported contribution	Microbiota-mediated repair support	(Yang et al., 2026b)

Original preparation names are retained; labels such as EVs or ELNs do not independently verify extracellular biogenesis. Outcomes refer to the tested preparation and should not be reassigned to *bona fide* extracellular vesicles without origin-specific evidence. Evidence categories describe the nature and depth of repair-related findings and are not a validated grading system. Experimental systems listed are those relevant to the summarized claim, not an exhaustive inventory of every assay. ‘*In vivo* with mechanistic assays’ does not imply a separately verified epithelial-cell efficacy model. Ex vivo-derived organoid cultures are cultured systems derived from isolated tissue. Increased proliferation/Lgr5 markers alone do not prove durable ISC regeneration. BMDMs, bone marrow-derived macrophages; PMA, phorbol ester; DSS, dextran sulfate sodium; TNBS, trinitrobenzenesulfonic acid; FMT, fecal microbiota transplantation; IPA, indole-3-propionic acid; IAA, indole-3-acetic acid; SCFAs, short-chain fatty acids. Administration entries do not imply oral delivery unless the oral route is explicitly identified. IECs, intestinal epithelial cells; ISCs, intestinal stem cells; DCs, dendritic cells; NETs, neutrophil extracellular traps; ILC3, group 3 innate lymphoid cells; TEER, transepithelial electrical resistance; AhR, aryl hydrocarbon receptor.

**FIGURE 2:**
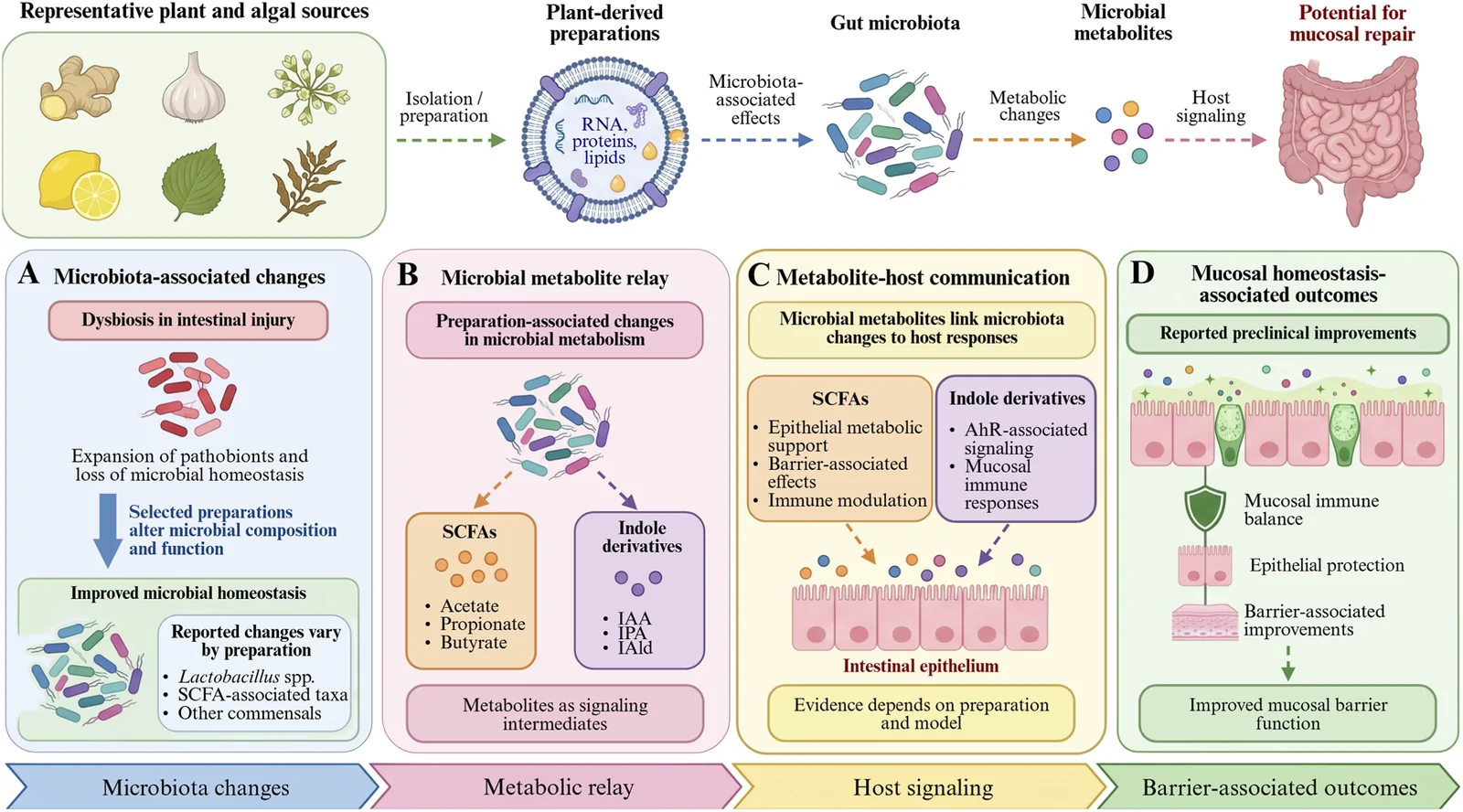
Microbiota-metabolite-host relay in plant-vesicle-associated intestinal mucosal repair. **(A)** Microbiota-associated changes. **(B)** Microbial metabolite relay. **(C)** Metabolite-host communication. **(D)** Mucosal homeostasis-associated outcomes. Study-specific particle labels do not establish extracellular biogenesis; microbial relays and causal evidence vary among preparations. Abbreviations: miRNA, microRNA; SCFAs, short-chain fatty acids; IAA, indole-3-acetic acid; IPA, indole-3-propionic acid; IAld, indole-3-carboxaldehyde; AhR, aryl hydrocarbon receptor; spp., species. Created in BioRender. liu, W. (2026) https://BioRender.com/4kab3uc.

## 6 Engineered therapeutic applications and delivery optimization of plant-derived carriers

Native plant-derived vesicular preparations exhibit varying degrees of gastrointestinal stability, biocompatibility, and cellular uptake. Drug loading and further engineering can build on these intrinsic properties to improve cargo stability, local exposure, and functional delivery efficiency (Zhang L. et al., 2026; Li Y. et al., 2024; Jung et al., 2025). Plant vesicle engineering is therefore evolving from the use of natural bioactivity alone toward drug loading, optimization of oral delivery, and enhancement of targeted and intracellular delivery.

### 6.1 Drug and nucleic acid delivery using plant-derived carriers

Plant-derived vesicular preparations and reconstructed lipid carriers can accommodate both hydrophobic small molecules and nucleic acids for local intestinal delivery (Barzin et al., 2023; Kürtösi et al., 2024). *Paederia scandens*-derived ELNs loaded with andrographolide improved drug dispersibility, stability, uptake, and experimental anti-colitis activity (Peng et al., 2025). *Callicarpa nudiflora* EVLPs loaded with budesonide similarly increased cellular uptake and local anti-inflammatory activity (Xi et al., 2026). These formulations combine carrier-associated activity with loaded therapeutics; contributions from the carrier, drug, and formulation should be assessed separately before claiming synergy.

Engineering extends plant-derived carriers to nucleic acid delivery. Reconstructed ginger lipids were used to deliver CD98 siRNA with colon targeting and gene silencing (Zha et al., 2017). Thermally reconstructed ginger particles supported TNF-α siRNA delivery and increased uptake by intestinal epithelial cells (Hou et al., 2026). A ginger membrane–ZIF-8 hybrid platform improved TNF-α siRNA loading, protection, and intracellular release (Cui et al., 2024). These reconstructed or hybrid products should not be equated with intact source vesicles. Evaluation should address RNA stability, target-cell uptake, intracellular release, target-gene silencing, and final tissue outcomes, in addition to encapsulation.

Plant-derived vesicular preparations as therapeutic carriers should therefore be evaluated not only in terms of loading capacity but also in terms of local intestinal exposure, target modulation, and final mucosal-repair outcomes.

### 6.2 Gastrointestinal protection and local colonic exposure during oral delivery

To be effective, orally administered plant-derived vesicular preparations must have sufficient stability in gastric acid, digestive enzymes and bile salts and must provide effective exposure in the targeted intestinal segment. Intrinsic gastrointestinal stability of plant sources will vary and formulation-based protection and site-specific delivery optimization may be important.

An enteric-coated formulation is a direct approach to increase gastrointestinal stability. The cabbage-derived plant vesicles are significantly disrupted in simulated upper gastrointestinal conditions; lyophilization, coating with Eudragit S100, and capsule formulation improve delivery of intact vesicles to the colon (Liu et al., 2023). Likewise, mRNA-loaded preparations derived from sweet orange can be lyophilized and formulated in acid-resistant capsules to improve nucleic-acid stability during oral delivery (Pomatto et al., 2023).

Upon arrival in the colon, hydrogels and polysaccharide-based materials can also be used to further control release and retention. Banana-derived nanovesicular preparations containing curcumin encapsulated by pectin–alginate hydrogel microspheres have shown good payload stability and improved release under colonic conditions (Liu H. et al., 2025). A formulation that combines vesicles made with extracts from American ginseng and hyaluronic-acid hydrogel with 5-ASA enhances topical treatment and mucosal repair (Weng et al., 2025). Rhubarb-based nanovesicles co-loaded with kaempferol in a chitosan/HP-β-CD hydrogel enable pH-responsive co-delivery to the colon and enhance local therapeutic effects (Tang et al., 2025). In another multistage strategy, ginger-derived GDNPs are loaded with celastrol and then embedded in a glycyrrhizic-acid hydrogel to form Cel-GDNPs@Gel, improving colonic retention, mucus penetration, and cellular uptake (Li N. et al., 2026).

In addition to protection by exogenous materials, some native plant-derived vesicular preparations show favorable gastrointestinal stability and retention within inflamed colonic tissue. *Momordica charantia*-derived MCEVs maintain particle integrity under simulated gastrointestinal conditions and exhibit prolonged retention in the inflamed colon after oral administration (Gao et al., 2025). A CX5461-loaded ELNV system derived from *Sophora flavescens* similarly shows good gastrointestinal stability and localization to inflamed tissue (Zhang et al., 2024). Curcumin-loaded ginger nanovesicles also preferentially distribute to the colon after oral administration, suggesting that cargo–carrier combinations may themselves influence local exposure (Huang et al., 2024).

Gastrointestinal protection, colonic targeting, and local retention collectively determine effective exposure after oral plant nanovesicle administration. Future delivery studies should assess the actual arrival of intact particles or functional cargo, persistence of exposure, and target-cell responses, rather than equating tissue fluorescence accumulation with effective delivery.

### 6.3 Enhanced targeting and intracellular delivery engineering

Once plant-derived vesicular preparations have reached the desired intestinal segment, they can be further engineered to improve recognition of diseased tissue, uptake by target cells, and intracellular cargo release. Surface-ligand modification is a relatively straightforward method. Hyaluronic acid (HA) modification of red cabbage-derived nanovesicular preparations enhances targeting efficiency toward intestinal epithelial and immune cells and improves therapeutic efficacy in colitis (Kang et al., 2024). Similar targeting approaches have been shown to be effective in other types of plant-derived vesicular preparations: folate targeting of lipid nanocarriers derived from grapefruit results in enhanced folate receptor-mediated uptake (Wang et al., 2013), while peptide modification of tumor-selective nanovesicles derived from turmeric yields a higher uptake by colorectal cancer cells (Meng et al., 2025).

In addition to ligand modification, protection of the cargo and release inside the cells could be enhanced using interfacial engineering and hybrid nanosystems. Polydopamine-based interfacial modification can alter surface properties and improve drug retention while enabling acid-responsive cargo release from plant-derived exosome-like particles (Khosravani et al., 2026). Lemon-derived particle-based oral capsules loaded with capecitabine enhanced colorectal tumor drug delivery through transient, mechanical-stress-associated opening of the intestinal epithelial barrier (Hao et al., 2026). This cancer-delivery mechanism should be distinguished from mucosal barrier restoration. Multistage delivery may be achieved by hybridizing plant-derived vesicular preparations with inorganic materials, thereby combining the biological recognition properties of natural membranes with the high loading capacity and controlled intracellular release enabled by synthetic materials. Functional peptide modifications like cRGD and TAT have also been demonstrated to improve cellular selectivity and uptake (Kang et al., 2026; Kanai et al., 2026). While some of this evidence is from tumor or non-intestinal models, principles of ligand recognition, cell penetration and stimulus-responsive design offer translatable strategies for engineering therapies for the inflamed intestinal mucosa (Yi et al., 2025; Wu et al., 2025; Liu et al., 2026). Table 5 summarizes representative delivery and engineering strategies, including drug and nucleic acid loading, gastrointestinal protection, hydrogel-assisted delivery, intrinsic colonic retention, surface-ligand modification, and interfacial engineering.

**TABLE 5:** Representative delivery and engineering strategies for plant-derived intestinal therapy.

Plant source	Strategy	Cargo/modification	Purpose and reported outcome	Limitation/evaluation focus	Refs.
*Paederia scandens*	Small-molecule loading	Andrographolide-loaded ELNs	Improved dispersion, stability, uptake, and experimental anti-colitis activity	Separate carrier and loaded-drug effects	(Peng et al., 2025)
*Callicarpa nudiflora*	Small-molecule loading	Budesonide-loaded EVLPs	Improved cellular uptake and local anti-inflammatory activity	Long-term repair evidence remains limited	(Xi et al., 2026)
Ginger	siRNA delivery	Reassembled source lipids with CD98 siRNA	Colon targeting and target-gene suppression	Measure intracellular release and sustained silencing	(Zha et al., 2017)
	Thermal reassembly	Reassembled particles with TNF-α siRNA	Increased IEC uptake and gene-silencing efficiency	Reassembly may alter native composition	(Hou et al., 2026)
	Hybrid inorganic carrier	Membrane–ZIF-8 with TNF-α siRNA	Improved RNA loading/protection and intracellular release	Additional materials complicate safety and manufacturing	(Cui et al., 2024)
	Hydrogel delivery	Celastrol-GDNPs in glycyrrhizic-acid hydrogel	Improved mucosal retention and local therapeutic exposure	Distinguish vesicle, gel, and drug contributions	(Li et al., 2026b)
	Cargo-carrier combination	Curcumin-loaded nanovesicles	Predominant colonic distribution after oral exposure	Cargo-dependent distribution needs characterization	(Huang et al., 2024)
Cabbage	Enteric protection	Freeze-drying, Eudragit S100 and capsules	Increased non-disrupted particles reaching the colon	Verify intact particles and functional cargo	(Liu et al., 2023)
Sweet orange	Gastric protection	mRNA particles in acid-resistant capsules	Improved oral nucleic-acid protection	Vaccine-delivery model, not direct mucosal-repair evidence	(Pomatto et al., 2023)
Banana	Hydrogel encapsulation	Curcumin vesicles in pectin–alginate beads	Improved stability and colon-responsive release	Quantify release and local potency	(Liu et al., 2025c)
American ginseng	Local hydrogel delivery	Vesicles, HA hydrogel and 5-ASA	Improved local treatment and mucosal healing	Multicomponent attribution remains difficult	(Weng et al., 2025)
Rhubarb	pH-responsive co-delivery	Nanovesicles and kaempferol in chitosan/HP-β-CD	Improved colonic delivery and combined effect	Batch consistency and long-term safety	(Tang et al., 2025)
*Momordica charantia*	Intrinsic local retention	MCEVs (original study label)	Gastrointestinal stability and prolonged inflamed-colon retention	Not itself an engineered formulation; rigorous integrity tracking required	(Gao et al., 2025)
*Sophora flavescens*	Lesion localization	CX5461-loaded ELNVs	Gastrointestinal compatibility and inflammatory localization	Fluorescence is not proof of functional cargo delivery	(Zhang et al., 2024)
Red cabbage	Surface targeting	Hyaluronic-acid modification	Enhanced inflammatory-cell/tissue targeting and colitis protection	Verify receptor dependence	(Kang et al., 2024)
Grapefruit	Receptor-targeted carrier	Folate-modified reassembled source lipids	Enhanced folate-associated cellular uptake	Early delivery-engineering platform; not direct mucosal-repair evidence	(Wang et al., 2013)
Turmeric	Peptide targeting	Tumor-selective peptide	Increased colorectal cancer-cell uptake	Tumor efficacy cannot establish inflammatory repair	(Meng et al., 2025)
Plant preparations	Interfacial modification	Polydopamine	Improved cargo protection and acid-responsive release	Evidence includes non-intestinal cancer systems	(Khosravani et al., 2026)
Lemon	Engineered oral capsules	Source-particle-based formulation	Transiently increased epithelial permeability/drug transport	Barrier opening in cancer delivery is not barrier repair	(Hao et al., 2026)
Various sources	Functional peptides	cRGD/TAT	Improved selective uptake or penetration	Mostly tumor/non-intestinal evidence	(Kang et al., 2026; Kanai et al., 2026)

Reconstructed lipid/membrane carriers are distinguished from intact native preparations. Intrinsic colonic retention is included as a complementary delivery property. Original EV labels describe the cited publications and do not independently establish extracellular biogenesis. HA, hyaluronic acid; ZIF-8, zeolitic imidazolate framework-8; HP-β-CD, hydroxypropyl-β-cyclodextrin; 5-ASA, 5-aminosalicylic acid; cRGD, cyclic Arg-Gly-Asp; TAT, trans-activator of transcription peptide.

As a whole, plant vesicle engineering could be considered as a sequence of delivery steps, beginning with cargo loading, followed by gastrointestinal protection, localization to the colon, targeted uptake, intracellular delivery, and functional modulation of molecular targets (Figure 3). It is no longer simply about increasing tissue accumulation; rather, the goal is lesion recognition, cellular uptake, and functional intracellular delivery. Future designs should focus on optimizing the step that currently limits therapeutic efficacy and should validate engineering benefits through target modulation and sustained mucosal repair. While these strategies significantly broaden the delivery capabilities for plant-derived vesicular preparations, the additional complexity of these formulations also represents additional translational challenges in areas of product consistency, mechanistic validation, safety, and scalable manufacturing.

**FIGURE 3:**
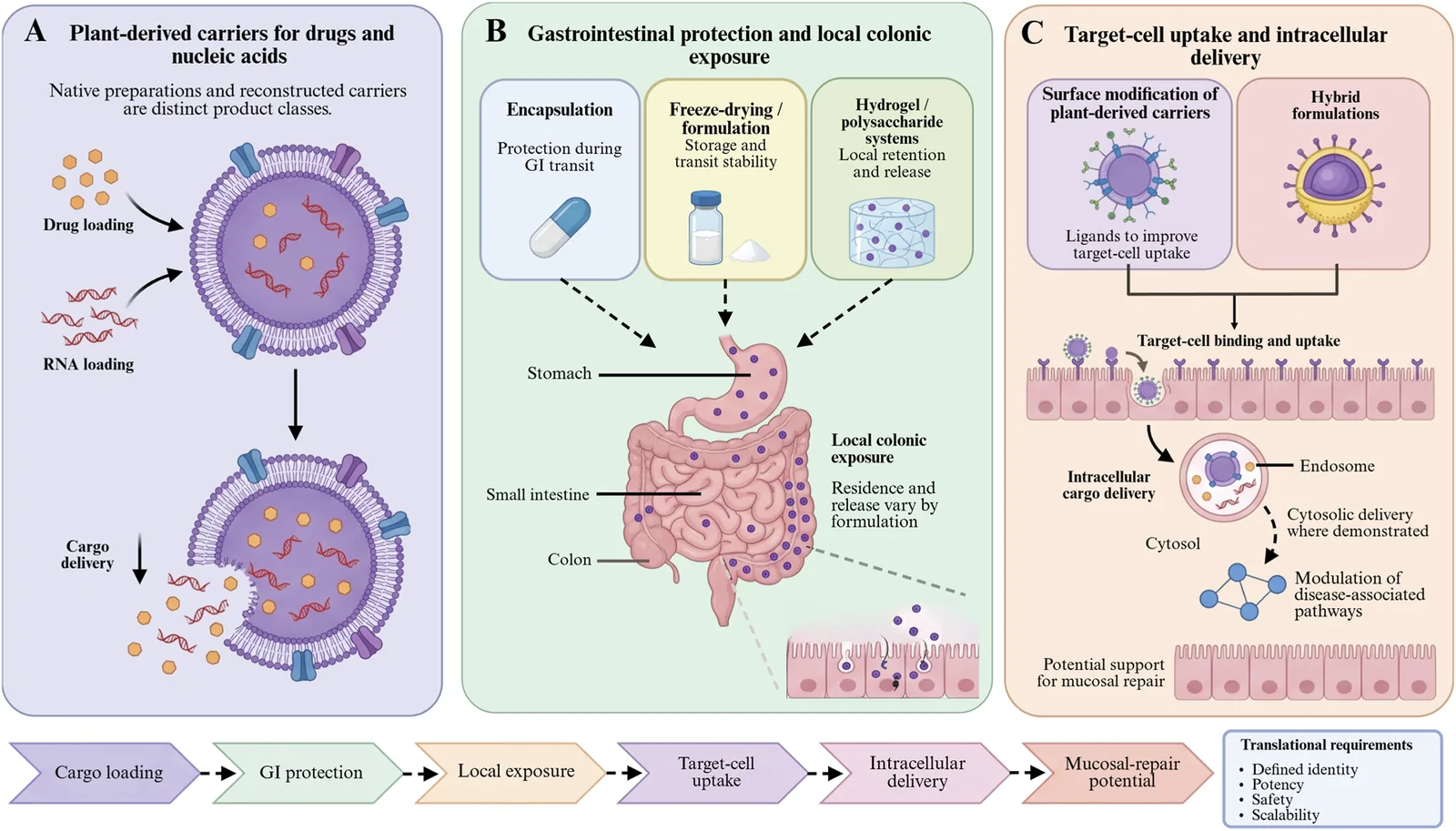
Engineering strategies for oral delivery of plant-derived vesicular preparations. **(A)** Drug and nucleic acid loading. **(B)** Gastrointestinal protection and colonic delivery. **(C)** Targeting and intracellular delivery. Native preparations and reconstructed lipid/membrane carriers are separate product classes and should be characterized accordingly. Abbreviations: RNA, ribonucleic acid; GI, gastrointestinal. Created in BioRender. liu, W. (2026) https://BioRender.com/nf7svqv.

## 7 Current limitations and challenges to clinical translation

### 7.1 Standardization and quality control

Product specifications should record plant species, tissue, source compartment, and processing/isolation methods, distinguishing extracellular-origin evidence from vesicle morphology (Alsaid et al., 2026; Welsh et al., 2024; Lian et al., 2022; Rodríguez de Lope et al., 2025). Native preparations and reconstructed carriers require separate characterization. Raw-material and processing variability can alter composition and potency, so a shared plant source does not establish product equivalence.

Beyond morphology, size, and surface charge (Rodríguez de Lope et al., 2025), quality control should assess membrane integrity, composition, purity, active cargo, biological potency, and batch consistency using defined dose metrics (Welsh et al., 2024; Shu et al., 2025; Yu et al., 2026; Bettin et al., 2023). Mechanism-linked cargo measurements should be coupled to functional potency assays (Welsh et al., 2024; Shu et al., 2025; Nguyen et al., 2020). Membrane lipid order has been investigated as a within-lot stability indicator in human fibroblast-derived EVs, but requires validation in plant-derived preparations (Tamura and Takeoka, 2026).

### 7.2 Limitations of preclinical evidence and mechanistic validation

Most plant nanovesicle research is still based on small-animal models, including DSS-induced colitis (Perše, 2026). Future studies should include more diverse models of chronic, genetic, immune-mediated, diet-associated, and infection-associated diseases, and incorporate more human-relevant systems, including patient-derived organoids, gut-on-chip platforms, and *ex vivo* human intestinal mucosa (Flood et al., 2024; Özkan et al., 2024).

Causal validation requires genetic or pharmacological perturbation, cargo depletion or gain-/loss-of-function, cell-population or microbiota manipulation, and rescue experiments linking active constituents to repair outcomes (Lv et al., 2025; Yang Y. et al., 2025; Luo et al., 2025; Somiya, 2020). Drawing on the broader EV field, studies should distinguish particle and cargo pharmacokinetics and connect dose, gastrointestinal stability, local exposure, uptake, intracellular delivery, target modulation, and biological effect (Gupta et al., 2021). Recent studies address biodistribution (Na et al., 2026), intestinal transport (Huang et al., 2026), and oral delivery (Zheng et al., 2026), but integrated quantitative dose–exposure–response relationships remain insufficiently established (Na et al., 2026; Huang et al., 2026; Gupta et al., 2021).

### 7.3 Safety, scalable manufacturing, and clinical translation

Edible origin does not establish pharmaceutical safety. Source materials may introduce pesticides, microbial contaminants, allergens, or unintended bioactive compounds, while therapeutic exposure differs from dietary consumption. Biodistribution, immunogenicity, repeat-dose toxicity, and long-term safety therefore require systematic evaluation (Na et al., 2026; Qiang et al., 2024; Chen and Xiao, 2026).

Raw-material, harvesting, storage, and processing variability can affect yield, composition, purity, and potency (Wang et al., 2022). Scalable purification, including TFF and chromatography, must balance recovery with integrity and batch consistency. Engineered formulations also require assessment of manufacturing complexity, batch comparability, and regulatory classification (Wang X. et al., 2026).

Broader EV experience, including osteoarthritis therapies, offers lessons on critical quality attributes, potency, and scalable production (Li and Toh, 2026), but does not constitute clinical validation for plant-derived intestinal therapies.

Clinical translation requires evidence of efficacy, biodistribution, and safety, together with justified indications, routes, doses, and schedules (Zhang L. et al., 2026; Chen and Xiao, 2026; Ning et al., 2025). Patient-centered development should specify the intended disease phenotype and distinguish clinical, endoscopic, biomarker, histological, and experimental barrier endpoints (Neurath and Vieth, 2023; Turner et al., 2021; Vieujean et al., 2026; Long, 2025). Short-term improvement in mouse colitis alone does not establish clinical utility.

## 8 Conclusions and perspectives

Plant-derived vesicular preparations show preclinical potential for immune regulation, epithelial protection, barrier-associated improvement, and microbiota–host communication. Evidence is strongest for selected anti-inflammatory and barrier-associated effects, whereas durable ISC-mediated regeneration and clinical mucosal healing remain insufficiently established.

Most intestinal studies evaluate heterogeneous ELN/PDNP preparations. Extracellularly released vesicles, tissue-disruption-derived particles, and reconstructed or hybrid carriers should remain distinct product classes; activity demonstrated for one preparation cannot be generalized to another. This distinction preserves foundational contributions while improving interpretation and reproducibility.

Translation requires defined product identity, reproducible potency, causal cargo validation, quantitative exposure–response relationships, scalable manufacturing, and long-term safety. Studies should integrate inflammatory, structural, regenerative, and functional outcomes and test durability after treatment withdrawal. These requirements will determine whether experimental benefits can support clinically useful mucosal-repair platforms.
